# New Insight into the Angle Insensitivity of Ultrathin Planar Optical Absorbers for Broadband Solar Energy Harvesting

**DOI:** 10.1038/srep32515

**Published:** 2016-09-01

**Authors:** Dong Liu, Haitong Yu, Yuanyuan Duan, Qiang Li, Yimin Xuan

**Affiliations:** 1 School of Energy and Power Engineering, Nanjing University of Science & Technology, Nanjing 210094, China; 2 Key Laboratory of Thermal Science and Power Engineering of Ministry of Education, Beijing Key Laboratory for CO2 Utilization and Reduction Technology, Tsinghua University, Beijing 100084, China; 3 School of Energy and Power Engineering, Nanjing University of Aeronautics & Astronautics, Nanjing 210016, China

## Abstract

Two challenging problems still remain for optical absorbers consisting of an ultrathin planar semiconductor film on top of an opaque metallic substrate. One is the angle-insensitive mechanism and the other is the system design needed for broadband solar energy harvesting. Here, first we theoretically demonstrates that the high refractive index, instead of the ultrathin feature as reported in previous studies, is the physical origin of the angle insensitivity for ultrathin planar optical absorbers. They exhibit omnidirectional resonance for TE polarization due to the high complex refractive index difference between the semiconductor and the air, while for TM polarization the angle insensitivity persists up to an incident angle related to the semiconductor refractive index. These findings were validated by fabricating and characterizing an 18 nm Ge/Ag absorber sample (representative of small band gap semiconductors for photovoltaic applications) and a 22 nm hematite/Ag sample (representative of large band gap semiconductors for photoelectrochemical applications). Then, we took advantage of angle insensitivity and designed a spectrum splitting configuration for broadband solar energy harvesting. The cascaded solar cell and unassisted solar water splitting systems have photovoltaic and photoelectrochemical cells that are also spectrum splitters, so an external spectrum splitting element is not needed.

Efficient optical absorbers with ultrathin planar structures are important for optoelectronic applications, such as photovoltaic (PV) and photoelectrochemical (PEC) cells. Solar cells with ultrathin semiconductor layers should have near 100% internal quantum efficiency because of the low electron-hole recombination losses resulting from the semiconductor thickness being much smaller than its exciton diffusion length^1^. The ultrathin feature also lowers the solar cell material and processing costs since around 40% of the cost is from the semiconductor wafer (for crystalline silicon)^2^. In addition, the fabrication costs are lower for devices with simple planar structures. Dotan *et al*.^3^ reported a high photocurrent density of 3.02 mA cm^−2^ at 1.6 V against the reversible hydrogen electrode in a PEC cell. They used a photoanode with an approximately 20 nm thick hematite film coated on a silver-gold alloy electron collection substrate. The photoanode had low bulk recombination losses due to the ultrathin film that corresponded to the hole diffusion length in hematite^4^,^5^,^6^. This photoanode also had lower surface and interface recombination losses compared with photoanodes using nanophotonic structures^7^,^8^,^9^ because of its planar design and resulting low surface areas of the hematite layer and the interfaces between the hematite and the electron collection layers^10^,^11^. Thus, ultrathin planar optical absorbers can significantly enhance system performance and reduce material and fabrication costs.

Multilayer films without any micro/nanoscale patterns have been widely used due to their translational invariance and the resultant resonance excited for a particular incident angle and wavelength. This well-defined resonance strongly depends on the incident angle for conventional optical absorbers based on lossless dielectric materials^12^. Omnidirectional plasmonic resonance was reported in Shin *et al*.^13^ and Lee *et al*.^14^ using a planar metal-insulator-metal structure, but this structure only works for the transverse-magnetic (TM) polarization. Kats *et al*.^12^ recently demonstrated enhanced optical absorption in an ultrathin planar lossy dielectric film coated on a metallic substrate (the structure was also used in Dotan *et al*.^3^) with high absorption for incident angles up to 60° for both transverse-electric (TE) and TM polarizations. They and Song *et al*.^15^ attributed the omnidirectional behavior to the ultrathin feature in their optical absorbers and estimated that “as these coatings are much thinner than the wavelength of light, there is little phase accumulation due to the propagation through the film compared with the reflection phase change on reflection.” However, Lee *et al*.^16^ and Park *et al*.^17^ found that the angle insensitivity resulted from the specific relationship between the propagation phase in the lossy medium and the reflection phase change from the metal. Therefore, further insight into the angle insensitivity is still needed.

Previous studies, to absorb a broad spectrum of the solar energy, have used either a vertical tandem cell configuration requiring a very sophisticated interface or have split the solar spectrum into multiple bands using a spectrum splitter, filter or prism^18^ which makes the systems more complex. PEC cells with hematite photoanodes require an external electrical bias to complete the water splitting reaction because hematite has a low conduction band edge below the reversible hydrogen potential^5^. Practically, unassisted PEC hydrogen production can be achieved using PEC-PV tandem cell configurations^19^,^20^,^21^,^22^,^23^ with perovskite or dye-sensitized solar cells providing the bias. In these configurations, the hematite photoanodes are usually semi-transparent, so photons with energies lower than the hematite band gap are transmitted through the photoanodes and absorbed by the solar cells. However, the ultrathin planar hematite photoanodes in Dotan *et al*.^3^ were opaque and thus, a PEC-PV cell with an external light splitting element might still be needed to drive unassisted solar hydrogen production, which complicates the entire system and increases system costs. Thus, this study is motivated to take advantage of the angle insensitivity of ultrathin planar optical absorbers to design new configurations for broadband solar energy harvesting.

Here, we experimentally and theoretically characterized an 18 nm Ge/Ag absorber (representative of small band gap semiconductors for photovoltaic applications) and a 22 nm hematite/Ag absorber (representative of large band gap semiconductors for photoelectrochemical applications), to demonstrate that the high refractive index is the physical origin of the angle insensitivity for absorbers consisting of an ultrathin planar semiconductor layer and an opaque metallic substrate. These absorbers show omnidirectional resonance for TE polarization due to the high refractive index difference between the semiconductor and air. For TM polarization, the angle insensitive regime is determined by the semiconductor refractive index and the resultant analogous Brewster angle. These findings were used to design a cascaded solar cell system and an unassisted solar water splitting system. In these systems, the photovoltaic and photoelectrochemical cells also work as spectrum splitters to utilize a broad range of the solar energy without an external spectrum splitting element.

## Results

### Experimental

**Figure 1:**
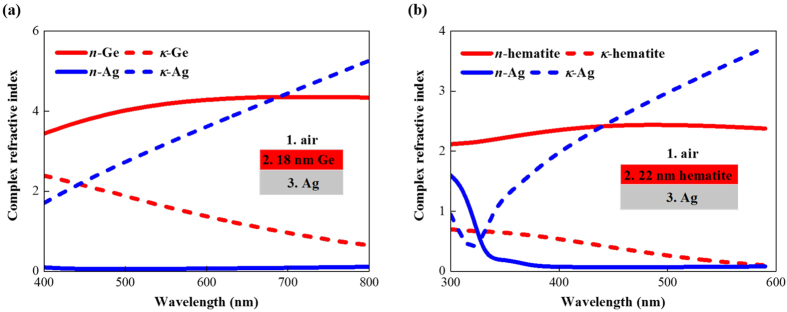
Complex refractive indices. (**a**) Ge and Ag deposited using electron beam evaporation and (**b**) hematite and Ag deposited using sputtering.

Optical absorbers were designed with an approximately 20 nm thick (an order of magnitude smaller than the visible wavelength) semiconductor layer coated on a silver (Ag) substrate as shown in the inset of Fig. 1. Two typical semiconductor materials were considered. Germanium (Ge) is representative of small band gap (<1.5 eV) semiconductors with refractive indices larger than 4 (such as silicon, III-V compounds). These semiconductors are usually used for PV applications. Hematite is representative of large band gap (>2.0 eV) semiconductors with refractive indices of 2 to 3 (such as metal oxides and sulfides). These semiconductors are usually used for PEC applications. An ultrathin Ge film was deposited on a polished silicon wafer using electron beam evaporation. The thickness and complex refractive index of this ultrathin Ge film were measured using the ellipsometry method. The measured thickness was 18 nm and the complex refractive index is shown in Fig. 1a. An ultrathin hematite film, deposited using sputtering, was characterized using the same method with a measured thickness of 22 nm and the complex refractive index shown in Fig. 1b. The 18 nm Ge/150 nm Ag and 22 nm hematite/150 nm Ag structures were coated on polished silicon wafers. The near-normal and oblique reflectivities of these structures were measured in the Spectrophotometry Laboratory of National Institute of Metrology in China using the PerkinElmer Lambda 950 spectrophotometer equipped with the universal reflectance accessory. The expanded uncertainty is 0.5% for incident angles below 45°, 1% for 45°–50° and 2% for 50°–60°.

The reflectivity coefficient, *r*, of these three layered structures is expressed as^24^


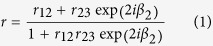


In Equation (1), for TE polarized light with incident angle *θ*_1_


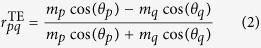


while for TM polarized light


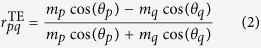


where *m*_*p*_ = *n*_*p*_ + *iκ*_*p*_ is the complex refractive index of layer *p* (*n*_*p*_ is the refractive index and *κ*_*p*_ is the extinction coefficient),


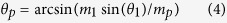



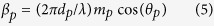


*d*_*p*_ is the thickness of medium *p* and *λ* is the wavelength. The spectral total reflectivity *R* = |r|^2^. Measured complex refractive indices were used in calculations (the measured complex refractive indices of Ag deposited using electron beam evaporation and sputtering are also shown in Fig. 1a,b respectively).

### Small band gap semiconductors

**Figure 2:**
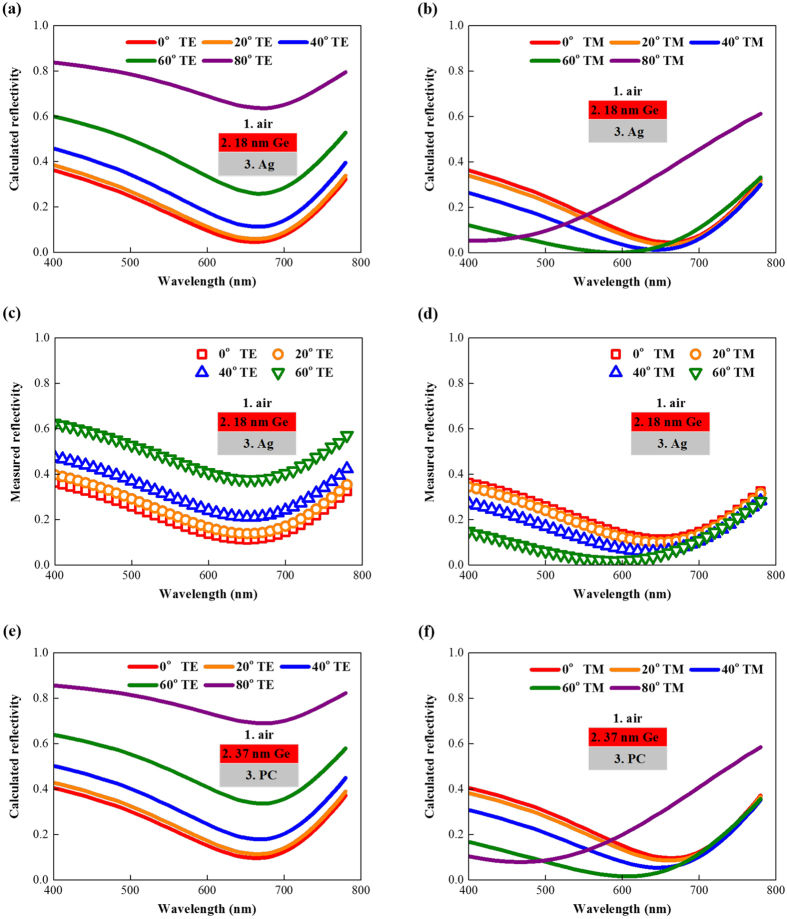
Optical properties of ultrathin planar absorbers based on small band gap semiconductors. (**a**) Calculated and (**c**) measured angular reflectivities of the 18 nm Ge/Ag structure for TE polarization and the (**b**) calculated and (**d**) measured results for TM polarization. Calculated angular reflectivities of 37 nm Ge/perfect electric conductor structure for (**e**) TE and (**f**) TM polarization.

The calculated reflectivities of the 18 nm Ge/Ag structure are shown in Fig. 2 for both the TE (Fig. 2a) and TM (Fig. 2b) polarizations for incident angles from 0° to 80°. The results show that this absorber achieves the Gires-Tournois resonance at 660 nm wavelength for normal incidence with the Ge thickness being only 1/8.5 of the resonant wavelength (*λ*/(8.5*n*_Ge_)) which illustrates the ultrathin feature. The results also show that the resonant wavelength is independent of the incident angle for TE polarization as shown in Fig. 2a, while for TM polarization the resonant wavelength is nearly constant for incident angles up to 40° and then shifts to shorter wavelengths for larger incident angles (Fig. 2b).

To determine whether these angular optical properties are caused by the ultrathin absorber thickness, we studied the absorber configuration with the Ag substrate replaced by a perfect electric conductor (PC) as shown in the inset of Fig. 2e. The angular reflectivity was calculated by setting *r*_23_ = −1 for TE polarization and *r*_23_ = 1 for TM polarization. To achieve resonance at 660 nm in this configuration, the Ge layer needed to be 37 nm thick which corresponds to a quarter wavelength. The results in Fig. 2e,f show that although this absorber is no longer ultrathin, it still has the same angular behavior as the ultrathin absorber.

This angular behavior was further understood by calculating the phase shift properties of these two absorber configurations. The Gires-Tournois resonant condition derived from Equation (1) is^25^,^26^


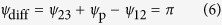


where *ψ*_12_ is the reflection phase shift at the air-Ge interface, *ψ*_p_ is the propagation phase shift in the Ge layer and *ψ*_12_ is the reflection phase shift at the Ge-substrate interface.

**Figure 3:**
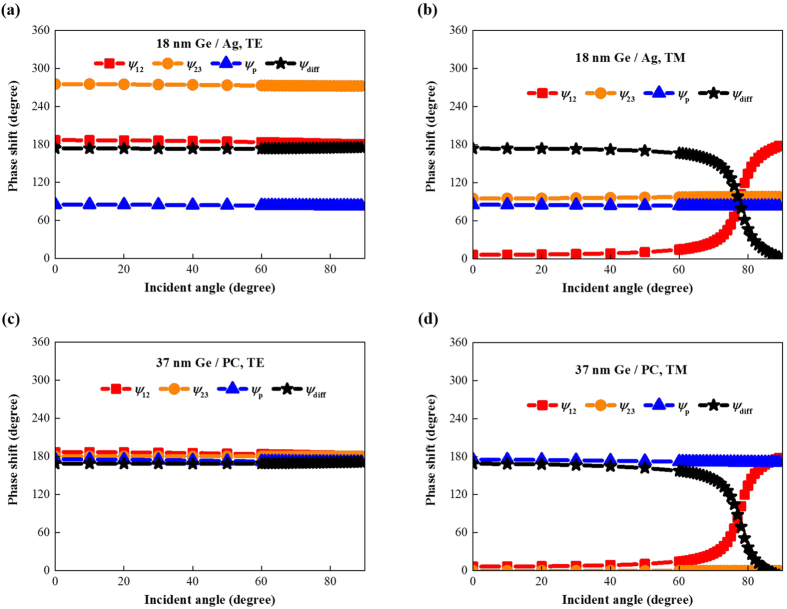
Angular phase shifts of ultrathin planar absorbers based on small band gap semiconductors. (**a**) TE and (**b**) TM polarizations for the 18 nm Ge/Ag structure. (**c**) TE and (**d**) TM polarizations for the 37 nm Ge/perfect electric conductor structure.

Figure 3a shows the phase shift results for the 18 nm Ge/Ag structure for TE polarization. We observe that *ψ*_12_ is around π while *ψ*_23_ is larger than π and, thus, *ψ*_p_, which is less than π, is enough to obtain a differential phase shift, *ψ*_diff_, of π, which means that the Ge layer can be thinner than a quarter wavelength. The reflection and propagation phase shifts of this structure are all independent of the incident angle; thus, the resultant angle-independent differential phase shift contributes to the omnidirectional resonance for TE polarization.

Figure 3b shows the results for TM polarization. *ψ*_12_ is around 0 for incident angles less than 60° but increases to near π for larger incident angles. *ψ*_23_ is independent of the incident angle and larger than 0. *ψ*_p_ is also angle independent and less than π. The resultant differential phase shift remains almost constant and equal to π below 60° but decreases for larger incident angles. This trend corresponds well to the angular reflectivity for TM polarization shown in Fig. 2b.

Figure 3c,d illustrate the results for the 37 nm Ge/PC structure for both polarizations. The results show that *ψ*_23_ is π for TE polarization and 0 for TM polarization due to the PC substrate. *ψ*_p_ is π corresponding to the quarter wavelength thickness needed to achieve resonance. The results also show that although the phase shift accumulated during propagation is as large as the reflection phase shifts picked up at the two interfaces, *ψ*_12_ and *ψ*_diff_ of this structure have the same angular variation as in the 18 nm Ge/Ag structure which shows that these two structures exhibit the same angular optical behavior.

Further insight into the physical origin of the angular behavior was gained by investigating the effects of the material properties, i.e. the complex refractive index, on the phase shift characteristics. Given that


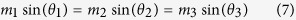


|*m*_2_| and |*m*_3_| are four times |*m*_1_|, and sin (*θ*_2_) and sin (*θ*_3_) are small, cos (*θ*_2_) and cos (*θ*_3_) must be near 1. Since *m*_2_ and *m*_3_ are complex, *θ*_2_ and *θ*_3_ are also complex and these quantities no longer simply represent the refraction angles. However, the analogy with the refraction phenomenon in lossless dielectrics is helpful. Due to the significant complex refractive index contrast between Ge and air, the analogous refraction angles *θ*_2_ and *θ*_3_ are small and almost independent of the incident angles.

For TE polarization, *ψ*_12_, the phase angle of 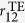, and *ψ*_23_ for 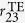 are expressed as


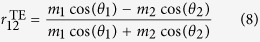



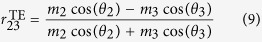


and *ψ*_p_ is the phase angle of 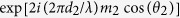. Equations (7) to (9), 10, 11 tell that *ψ*_12_, *ψ*_23_ and *ψ*_p_ are all independent of the incident angle. In addition, 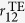 is near −1; thus, *ψ*_12_ is near π. These results agree well with results in Fig. 3a.

For TM polarization, *ψ*_12_, the phase angle of 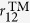, and *ψ*_23_ for 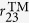 are expressed as


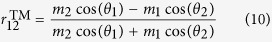



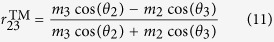


and *ψ*_p_ is the same as for TE polarization. Equations (7) to (9), 10, 11 tell that *ψ*_23_ and *ψ*_p_ are both independent of the incident angle while *ψ*_12_ changes with the incident angle. 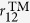 is near 1 for small incident angles and near −1 for large angles; thus, *ψ*_12_ increases from 0 to π. This change differs from the discontinuous change at the Brewster angle for lossless dielectrics. However, *ψ*_12_ does change sharply at the analogous Brewster angle determined by arctan(*n*_Ge_/*n*_air_). These results agree well with the results in Fig. 3b.

This analysis also applies to the 37 nm Ge/PC structure and agrees well with the results in Fig. 3c,d, which demonstrates again that the angular behavior of ultrathin planar optical absorbers have nothing to do with their ultrathin feature.

These theoretical results agree well with the measured reflectivities shown in Fig. 2c,d. The calculations accurately predict the resonant wavelength with the same angular dependence as the experimental data.

### Large band gap semiconductors

**Figure 4:**
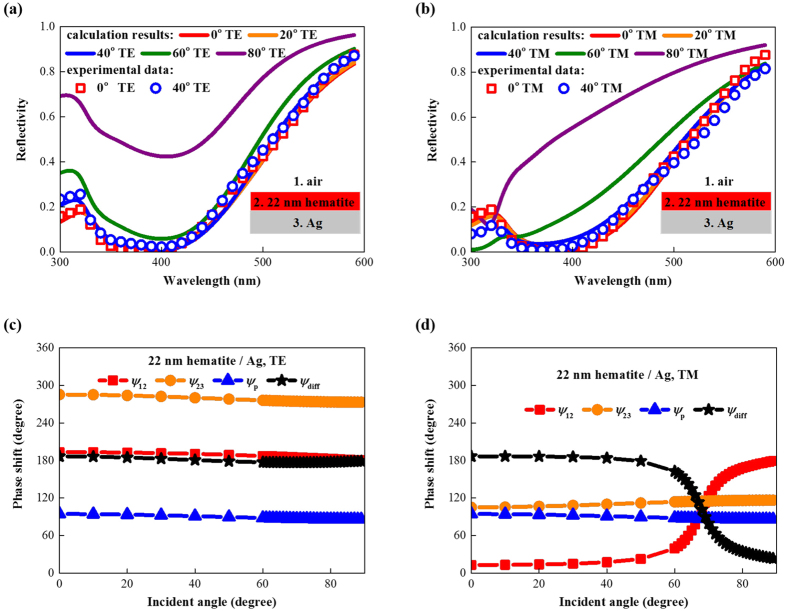
Optical properties of ultrathin planar absorbers based on large band gap semiconductors. Calculated and measured angular reflectivities of the 22 nm hematite/Ag structure for (**a**) TE and (**b**) TM polarizations. Angular phase shifts of this structure for (**c**) TE and (**d**) TM polarizations.

The angular reflectivities of the 22 nm hematite/Ag structure were calculated for both TE (Fig. 4a) and TM (Fig. 4b) polarizations. The results show that this low refractive index semiconductor absorber also exhibits omnidirectional resonance at 390 nm for TE polarization. Although the refractive index of hematite is only twice the index of air, simple calculations show that cos (*θ*_2_) is larger than 0.9 for all incident angles; thus, large band gap semiconductor absorbers have angular behavior similar to small band gap semiconductor absorbers. For TM polarized light with incident angles larger than 40°, the 22 nm hematite/Ag structure has more significant blue-shift of the resonant wavelength than the 18 nm Ge/Ag structure because of the smaller refractive index and resultant analogous Brewster angle. These results are validated by the experimental data in Fig. 4a,b and the phase shift results shown in Fig. 4c,d.

Materials with refractive indices less than 2 are generally insulators and cannot be used as active materials for PV and PEC applications, so these materials are not discussed here (see Supplementary Information for details).

### Applications for broadband solar energy harvesting

The previous sections showed that ultrathin planar optical absorbers based on both small and large band gap semiconductors exhibit omnidirectional resonance for TE polarization. For TM polarization the angle insensitivity persists up to incident angles near the analogous Brewster angle determined by the refractive index of the semiconductors. These findings can be used to estimate the incident angle range with omnidirectional resonance for unpolarized light, which is needed for efficient solar energy harvesting due to the unpolarized nature of sunlight^27^,^28^,^29^,^30^.

**Figure 5:**
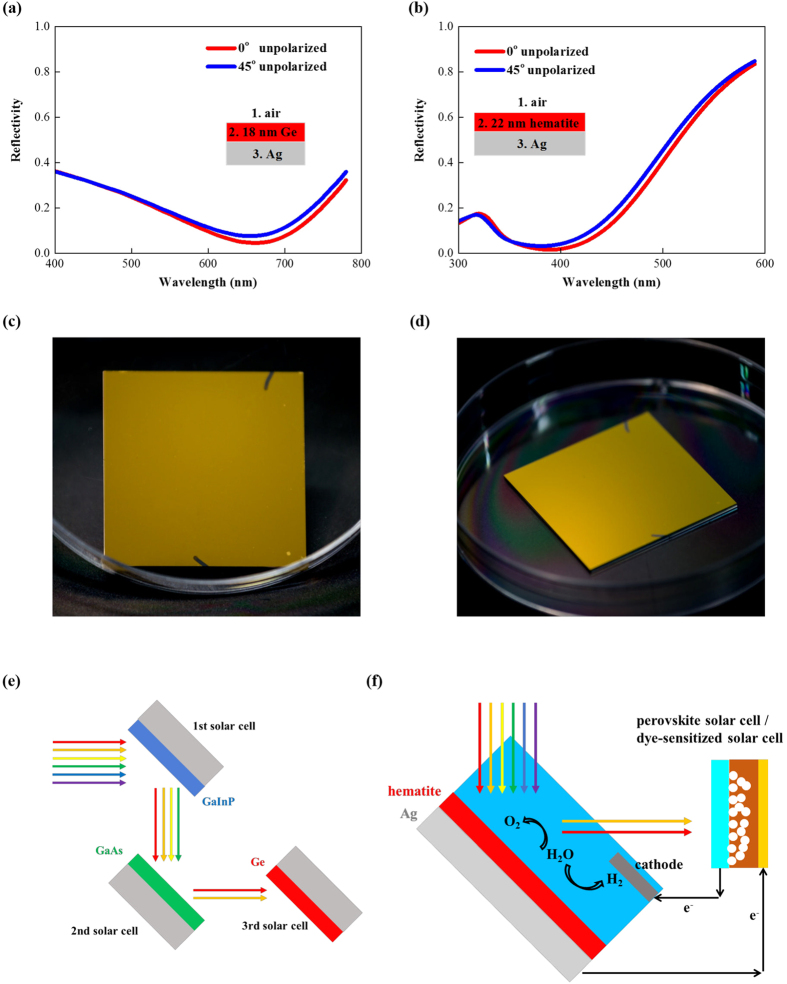
Spectrum splitting configurations. Reflectivity of (**a**) 18 nm Ge/Ag and (**b**) 22 nm hematite/Ag for normal and 45° incident unpolarized light. Photographs of the fabricated 22 nm hematite/Ag sample taken from (**c**) 0° and (**d**) 45° angles. (**e**) Cascaded solar cells and (**f**) unassisted solar water splitting.

Figure 5a,b show that the reflectivity for 45° incident unpolarized light is almost the same as for normal incidence with the peak absorptivity greater than 90% for both the 18 nm Ge/Ag and 22 nm hematite/Ag structures. Images of the fabricated 22 nm hematite/Ag sample were shown in Fig. 5c,d with the unchanged color clearly demonstrating the angle insensitivity. This angle insensitivity was then used to design a spectrum splitting configuration where the ultrathin planar optical absorber works as a spectrum splitter. The cascaded solar cell system shown in Fig. 5e was designed for small band gap semiconductor absorbers. The sunlight is obliquely incident on the first GaInP solar cell where the short-wavelength photons are absorbed. Longer wavelength photons are reflected and absorbed by the second GaAs cell. The third Ge cell then harvests the remaining photons. We designed an unassisted solar water splitting system as shown in Fig. 5f for large band gap semiconductor absorbers. The sun light is incident on the PEC cell at an oblique angle. The PEC cell efficiently absorbs photons in blue and green bands due to both the Gires-Tournois optical resonance and the angle insensitivity. Longer wavelength photons are reflected by the PEC cell and harvested by a PV cell (perovskite or dye-sensitized solar cells) to provide the required external electrical bias to drive the unassisted hydrogen production. In both systems, the PV and PEC cells also work as spectrum splitters, so an external spectrum splitting element is not needed. These configurations can also be applied to other systems targeting a broad solar energy spectrum such as photovoltaic-thermoelectric hybrid systems^31^.

## Discussion

In summary, we have demonstrated that the high refractive index contributes to the angle insensitivity for ultrathin planar optical absorbers consisting of an ultrathin semiconductor layer on top of an opaque metallic layer. This was validated theoretically and experimentally for an 18 nm Ge/Ag absorber (representative of small band gap semiconductors with refractive indices larger than 4 for PV applications) and a 22 nm hematite/Ag absorber (representative of large band gap semiconductors with refractive indices of 2 to 3 for PEC applications). These absorbers exhibit omnidirectional resonance for TE polarization due to the high refractive index difference between the semiconductor and air, with the angle insensitivity persisting up to an incident angle determined by the semiconductor refractive index for TM polarization. A spectrum splitting configuration was then designed to take advantage of this angle insensitivity to harness broadband solar energy. The photovoltaic cells in the cascaded solar cell system and the photoelectrochemical cells in the unassisted solar water splitting system also work as spectrum splitters; thus, external spectrum splitting elements are not needed. These configurations can also be used in other solar energy systems.

## Additional Information

**How to cite this article**: Liu, D. *et al*. New Insight into the Angle Insensitivity of Ultrathin Planar Optical Absorbers for Broadband Solar Energy Harvesting. *Sci. Rep.* **6**, 32515; doi: 10.1038/srep32515 (2016).

## Supplementary Material

Supplementary Information
