# Supplementary material for: New Insight into the Angle Insensitivity of Ultrathin Planar Optical Absorbers for Broadband Solar Energy Harvesting
Source: Sci Rep. 2016 Sep 1;6:32515. doi: 10.1038/srep32515 (PMC5007480; doi:10.1038/srep32515)
Supplement: Supplementary Information [file srep32515-s1.pdf]

Supplementary Information

New Insight into the Angle Insensitivity of Ultrathin Planar Optical Absorbers for Broadband Solar Energy Harvesting

Dong Liu[1, *], Haitong Yu[3], Yuanyuan Duan[3], Qiang Li[1], Yimin Xuan[1, 2]

[1]School of Energy and Power Engineering, Nanjing University of Science & Technology, Nanjing 210094, China

[2]School of Energy and Power Engineering, Nanjing University of Aeronautics & Astronautics, Nanjing 210016, China

[3]Key Laboratory of Thermal Science and Power Engineering of Ministry of Education, Beijing Key Laboratory for CO_2 Utilization and Reduction Technology, Tsinghua University, Beijing 100084, China

[*]Email address: liudong15@njust.edu.cn

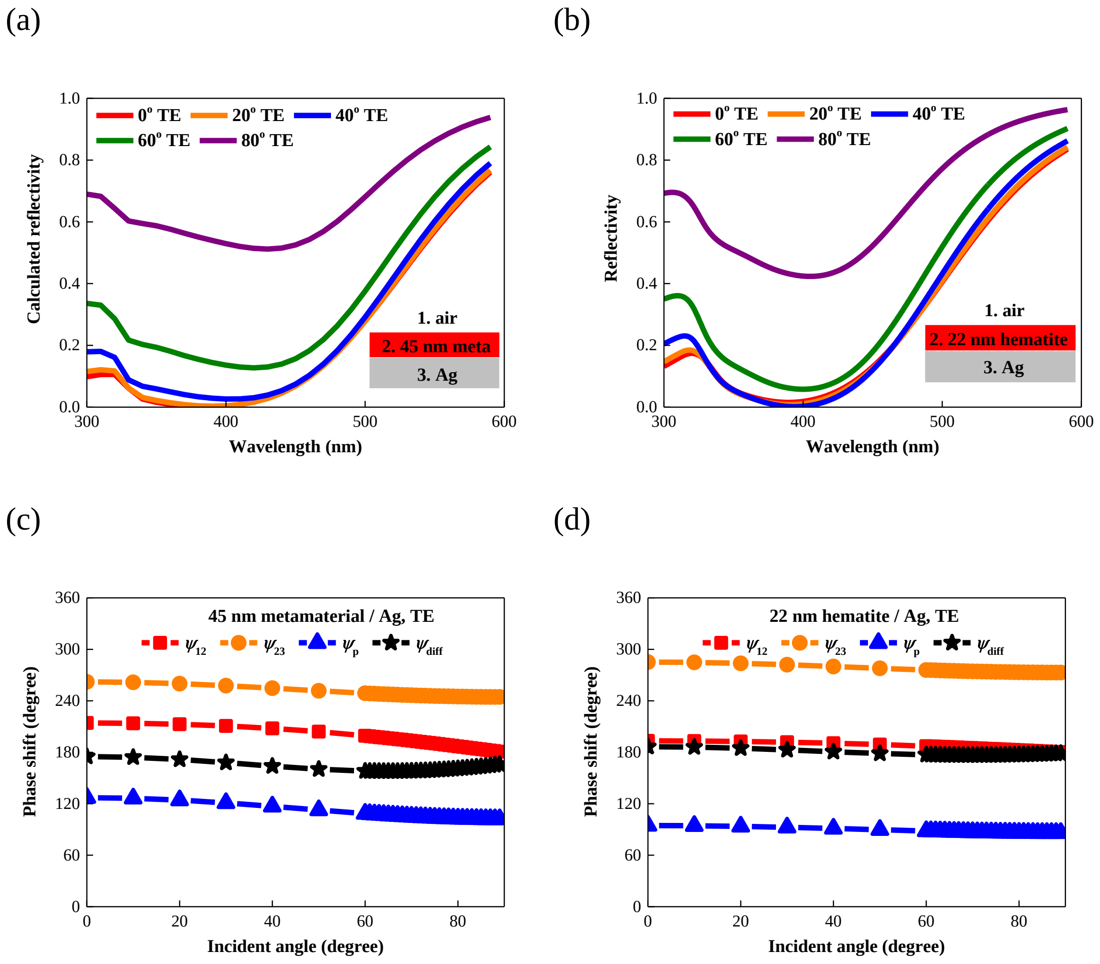

Figure S1. Optical properties of the ultrathin planar absorber based on a lossy material with low refractive index. Calculated angular reflectivity of (a) the 45 nm metamaterial/Ag structure and (b) the 22 nm hematite/Ag structure for TE polarization. Angular phase shifts of (a) the 45 nm metamaterial/Ag structure and (b) the 22 nm hematite/Ag structure for TE polarization.

To make our work more complete, we assumed a lossy “metamaterial” with low refractive index since materials with refractive indices less than 2 are generally insulators losing the lossy nature as we stated in the manuscript. Here, we assumed that $n_{meta}=n_{hematite}-0.8$ and $\kappa_{meta}=\kappa_{hematite}$ so the refractive index of this metamaterial is approximately 1.5.

The reflectivities of the 45 nm metamaterial/Ag structure were then calculated for the TE polarization for incident angles from 0° to 80° as shown in Figure S1(a). The results show that this absorber achieves the Gires-Tournois resonance at 390 nm wavelength for normal incidence with the metamaterial thickness being 1/6 of the resonant wavelength. The results also show that the resonant wavelength shifts more significantly to longer wavelengths for larger incident angles compared with the 22 nm hematite/Ag structure shown in Figure S1(b).

Figure S1(c) shows the phase shift results for the 45 nm metamaterial/Ag structure for TE polarization. We observe that ψ_{12}, ψ_{p} and the resultant ψ_{diff} all change more significantly with incident angles compared with the 22 nm hematite/Ag structure shown in Figure S1(d) which explains that the 45 nm metamaterial/Ag structure is more angle-sensitive.
